# 1,1-Diethyl-3-(4-meth­oxy­benzo­yl)thio­urea

**DOI:** 10.1107/S1600536811049208

**Published:** 2011-11-25

**Authors:** Aisha A. Al-abbasi, Mohamed Ibrahim Mohamed Tahir, Mohammad B. Kassim

**Affiliations:** a School of Chemical Sciences & Food Technology, Faculty of Science & Technology, Universiti Kebangsaan Malaysia, 43600 Bangi, Selangor, Malaysia; b Department of Chemistry, Faculty of Science, Universiti Putra Malaysia, 43400 UPM Serdang, Selangor, Malaysia; c Fuel Cell Institute, Universiti Kebangsaan Malaysia, 43600 Selangor, Malaysia

## Abstract

In the title compound, C_13_H_18_N_2_O_2_S, the 4-meth­oxy­benzoyl fragment is approximately planar [maximum deviation = 0.057 (2) Å] and twisted relative to the thio­amide fragment, forming a dihedral angle of 86.62 (6)°. The two C*sp* ^2^—N*sp* ^2^ bonds in the thio­urea unit differ significantly in length [1.327 (2) and 1.431 (2) Å]. In the crystal, N—H⋯O hydrogen bonds link the mol­ecules into chains parallel to [010].

## Related literature

For structural parameters and chemical properties of 1,1 disubstituted 3-benzoyl­thio­ureas, see: Al-abbasi *et al.* (2010 ▶, 2011 ▶); Al-abbasi & Kassim (2011 ▶); Mohamadou *et al.* (1994 ▶).
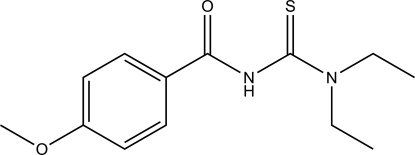


## Experimental

### 

#### Crystal data

C_13_H_18_N_2_O_2_S
*M* *_r_* = 266.35
Orthorhombic, 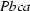

*a* = 12.9024 (5) Å
*b* = 10.0095 (4) Å
*c* = 20.8585 (11) Å
*V* = 2693.8 (2) Å^3^
*Z* = 8
Cu *K*α radiation
μ = 2.11 mm^−1^
*T* = 150 K
0.24 × 0.10 × 0.05 mm

#### Data collection

Oxford Diffraction Gemini diffractometer
Absorption correction: multi-scan (*CrysAlis RED*; Oxford Diffraction, 2006 ▶) *T*_min_ = 0.810, *T*_max_ = 0.900
12046 measured reflections
2548 independent reflections
2214 reflections with *I* > 2σ(*I*)
*R*_int_ = 0.030

#### Refinement

*R*[*F* ^2^ > 2σ(*F* ^2^)] = 0.042
*wR*(*F* ^2^) = 0.132
*S* = 1.12
2548 reflections
163 parameters
H-atom parameters constrained
Δρ_max_ = 0.43 e Å^−3^
Δρ_min_ = −0.32 e Å^−3^

### 

Data collection: *CrysAlis CCD* (Oxford Diffraction, 2006 ▶); cell refinement: *CrysAlis RED* (Oxford Diffraction, 2006 ▶); data reduction: *CrysAlis RED*; program(s) used to solve structure: *SHELXS97* (Sheldrick, 2008 ▶); program(s) used to refine structure: *SHELXL97* (Sheldrick, 2008 ▶); molecular graphics: *SHELXTL* (Sheldrick, 2008 ▶); software used to prepare material for publication: *SHELXTL*, *PLATON* (Spek, 2009 ▶) and *publCIF* (Westrip, 2010 ▶).

## Supplementary Material

Crystal structure: contains datablock(s) I, global. DOI: 10.1107/S1600536811049208/gk2435sup1.cif

Structure factors: contains datablock(s) I. DOI: 10.1107/S1600536811049208/gk2435Isup2.hkl

Supplementary material file. DOI: 10.1107/S1600536811049208/gk2435Isup3.cml

Additional supplementary materials:  crystallographic information; 3D view; checkCIF report

## Figures and Tables

**Table 1:** Hydrogen-bond geometry (Å, °)

*D*—H⋯*A*	*D*—H	H⋯*A*	*D*⋯*A*	*D*—H⋯*A*
N1—H1*A*⋯O1^i^	0.86	2.05	2.847 (2)	154

Symmetry code: (i) 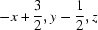.

**Fig. 1.**
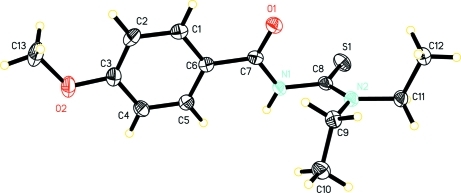
The molecular structure of 1,1-diethyl-3-(4-methoxybenzoyl)thiourea with displacement ellipsods drawn at the 50% probability level.

**Fig. 2.**
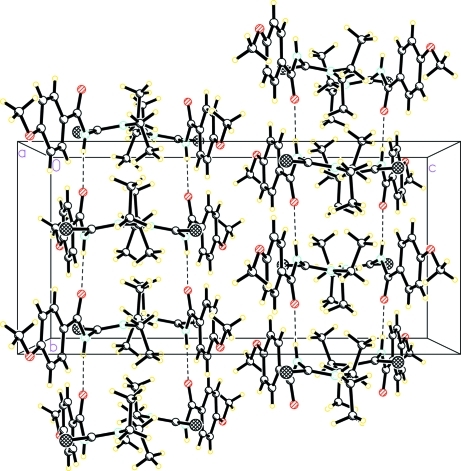
The crystal packing of 1,1-diethyl-3-(4-methoxybenzoyl)thiourea with intermolecular hydrogen bonds shown as dashed lines.

## supplementary crystallographic information

### Comment

The title compound (I) has been used as a ligand to form stable complexes with Ni and Co (Mohamadou *et al.*, 1994). Compund I and other similar derivatives act as a bidentate (*O,S*) chelate  forming square planar and tetrahedral complexes with Ni^II ^and Co^III^, respectively.

In the structure of   I, the 4-methoxybenzamide moiety [O2/N1/C1/C2/C3/C4/C5/C6/C7/C8/C13] (A) and the thiourea fragment [S1/N1/N2/C8] (B) are essentially planar with maximum deviations from the mean planes  0.057 (2) Å for C13 and -0.031 (2) Å N1. The dihedral angle between the A and B planes is 86.62 (6)°, which is slightly smaller than the analogous dihedral angle [87.99 (11)°] in 1-benzoyl-3-ethyl-3-phenylthiourea (II) (Al-abbasi & Kassim, 2011).

The C=O [1.237 (2) Å] and C=S [1.658 (3) Å] bond lengths are slightly longer than those in II [1.207 (3) and 1.666 (2) Å, respectively].

In the crystal, the molecules are stabilized by intermolecular N1—H1A···O1 hydrogen bonds forming a one-dimensional polymeric network along the *b* axis (Figure 2).

### Experimental

A solution of benzoyl chloride (10 mmol) in acetone was added slowly to an equimolar solution of ammonium thiocyanate in acetone. The reaction mixture was stirred at room temperature before adding diethylamine (10 mmol) slowly and the mixture was left stirring at room temperature for 2–3 h. The mixture was poured onto a water-ice, filtered and the residue was recrystallized from ethaaol/acetone solution to give colourless crystals, suitable for X-ray crystallography (yield 85%).

### Refinement

The hydrogen atom positions were calculated geometrically and refined in a riding model approximation with C–H bond lengths in the range 0.93–0.97 Å and N-H= 0.86 Å with *U*_iso_(H) = 1.2*U*_eq_(C, N) for N-H, aromatic C-H and CH_2_ groups, and *U*_iso_(H) = 1.5*U*_eq_(C) for methyl group.

### Crystal data

**Table:**

C_13_H_18_N_2_O_2_S	*D*_x_ = 1.314 Mg m^−^^3^
*M**_r_* = 266.35	Melting point = 407.15–408.15 K
Orthorhombic, *P**b**c**a*	Cu *K*α radiation, λ = 1.54178 Å
Hall symbol: -P 2ac 2ab	Cell parameters from 5648 reflections
*a* = 12.9024 (5) Å	θ = 4.2–71.1°
*b* = 10.0095 (4) Å	µ = 2.11 mm^−^^1^
*c* = 20.8585 (11) Å	*T* = 150 K
*V* = 2693.8 (2) Å^3^	Plate, colourless
*Z* = 8	0.24 × 0.10 × 0.05 mm
*F*(000) = 1136	

### Data collection

**Table:**

Oxford Diffraction Gemini diffractometer	2548 independent reflections
Radiation source: fine-focus sealed tube	2214 reflections with *I* > 2σ(*I*)
graphite	*R*_int_ = 0.030
ω/2θ scans	θ_max_ = 71.1°, θ_min_ = 4.2°
Absorption correction: multi-scan (*CrysAlis RED*; Oxford Diffraction, 2006)	*h* = −13→15
*T*_min_ = 0.810, *T*_max_ = 0.900	*k* = −12→12
12046 measured reflections	*l* = −23→25

### Refinement

**Table:**

Refinement on *F*^2^	Primary atom site location: structure-invariant direct methods
Least-squares matrix: full	Secondary atom site location: difference Fourier map
*R*[*F*^2^ > 2σ(*F*^2^)] = 0.042	Hydrogen site location: inferred from neighbouring sites
*wR*(*F*^2^) = 0.132	H-atom parameters constrained
*S* = 1.12	*w* = 1/[σ^2^(*F*_o_^2^) + (0.0835*P*)^2^ + 0.6035*P*] where *P* = (*F*_o_^2^ + 2*F*_c_^2^)/3
2548 reflections	(Δ/σ)_max_ < 0.001
163 parameters	Δρ_max_ = 0.43 e Å^−^^3^
0 restraints	Δρ_min_ = −0.32 e Å^−^^3^

### Special details

**Table:**

Experimental. The crystal was placed in the cold stream of an Oxford Cryosystems open-flow nitrogen cryostat (Cosier & Glazer, 1986) with a nominal stability of 0.1 K.Cosier, J. & Glazer, A.*M*., 1986. J. Appl. Cryst. 105 107.
Geometry. All e.s.d.'s (except the e.s.d. in the dihedral angle between two l.s. planes) are estimated using the full covariance matrix. The cell e.s.d.'s are taken into account individually in the estimation of e.s.d.'s in distances, angles and torsion angles; correlations between e.s.d.'s in cell parameters are only used when they are defined by crystal symmetry. An approximate (isotropic) treatment of cell e.s.d.'s is used for estimating e.s.d.'s involving l.s. planes.
Refinement. Refinement of *F*^2^ against ALL reflections. The weighted *R*-factor *wR* and goodness of fit *S* are based on *F*^2^, conventional *R*-factors *R* are based on *F*, with *F* set to zero for negative *F*^2^. The threshold expression of *F*^2^ > σ(*F*^2^) is used only for calculating *R*-factors(gt) *etc*. and is not relevant to the choice of reflections for refinement. *R*-factors based on *F*^2^ are statistically about twice as large as those based on *F*, and *R*- factors based on ALL data will be even larger.

### Fractional atomic coordinates and isotropic or equivalent isotropic displacement parameters (Å^2^)

**Table:**

	*x*	*y*	*z*	*U*_iso_*/*U*_eq_	
S1	0.96773 (4)	0.08865 (5)	0.60731 (2)	0.0302 (2)	
O1	0.72174 (10)	0.27396 (13)	0.63046 (7)	0.0282 (3)	
O2	0.29590 (10)	0.00839 (15)	0.54492 (7)	0.0327 (4)	
N1	0.76759 (11)	0.05775 (15)	0.63691 (7)	0.0210 (3)	
H1A	0.7495	−0.0246	0.6332	0.025*	
N2	0.87609 (11)	0.11414 (15)	0.72156 (7)	0.0215 (3)	
C1	0.52575 (14)	0.21406 (19)	0.58056 (9)	0.0242 (4)	
H1B	0.5472	0.3028	0.5811	0.029*	
C2	0.42448 (15)	0.1834 (2)	0.56253 (9)	0.0271 (4)	
H2A	0.3785	0.2510	0.5513	0.033*	
C3	0.39293 (14)	0.0509 (2)	0.56149 (8)	0.0246 (4)	
C4	0.46228 (14)	−0.05050 (19)	0.57806 (9)	0.0236 (4)	
H4A	0.4410	−0.1393	0.5771	0.028*	
C5	0.56228 (14)	−0.01898 (19)	0.59584 (8)	0.0220 (4)	
H5A	0.6082	−0.0870	0.6067	0.026*	
C6	0.59545 (14)	0.11370 (18)	0.59775 (8)	0.0201 (4)	
C7	0.69945 (13)	0.15522 (17)	0.62164 (8)	0.0202 (4)	
C8	0.86948 (13)	0.08980 (16)	0.65915 (9)	0.0213 (4)	
C9	0.78567 (13)	0.11498 (19)	0.76513 (9)	0.0236 (4)	
H9A	0.7265	0.1524	0.7427	0.028*	
H9B	0.8007	0.1721	0.8015	0.028*	
C10	0.75793 (15)	−0.0231 (2)	0.78918 (10)	0.0298 (4)	
H10A	0.6989	−0.0174	0.8171	0.045*	
H10B	0.8156	−0.0599	0.8123	0.045*	
H10C	0.7417	−0.0798	0.7534	0.045*	
C11	0.97664 (13)	0.14064 (18)	0.75210 (9)	0.0246 (4)	
H11A	1.0310	0.0973	0.7277	0.029*	
H11B	0.9768	0.1028	0.7949	0.029*	
C12	0.99913 (15)	0.28917 (19)	0.75623 (10)	0.0296 (5)	
H12A	1.0650	0.3028	0.7766	0.044*	
H12B	0.9459	0.3322	0.7808	0.044*	
H12C	1.0008	0.3265	0.7138	0.044*	
C13	0.22137 (16)	0.1077 (3)	0.52797 (11)	0.0401 (5)	
H13A	0.1567	0.0654	0.5177	0.060*	
H13B	0.2457	0.1569	0.4914	0.060*	
H13C	0.2116	0.1676	0.5634	0.060*	

### Atomic displacement parameters (Å^2^)

**Table:**

	*U*^11^	*U*^22^	*U*^33^	*U*^12^	*U*^13^	*U*^23^
S1	0.0251 (3)	0.0307 (3)	0.0347 (3)	−0.00535 (18)	0.00843 (18)	−0.00309 (18)
O1	0.0258 (7)	0.0211 (7)	0.0378 (8)	−0.0015 (5)	−0.0037 (6)	0.0012 (5)
O2	0.0193 (7)	0.0438 (9)	0.0351 (8)	−0.0006 (6)	−0.0042 (5)	0.0002 (6)
N1	0.0196 (7)	0.0170 (8)	0.0265 (8)	−0.0021 (5)	−0.0025 (6)	−0.0007 (6)
N2	0.0188 (7)	0.0181 (7)	0.0275 (8)	−0.0002 (5)	−0.0016 (6)	0.0003 (6)
C1	0.0273 (10)	0.0215 (10)	0.0238 (10)	0.0017 (7)	0.0004 (7)	0.0011 (7)
C2	0.0255 (9)	0.0315 (11)	0.0244 (9)	0.0085 (8)	−0.0015 (7)	0.0035 (7)
C3	0.0207 (9)	0.0348 (11)	0.0184 (9)	0.0003 (7)	0.0004 (6)	−0.0014 (7)
C4	0.0242 (9)	0.0227 (9)	0.0239 (10)	−0.0025 (7)	0.0016 (7)	−0.0026 (7)
C5	0.0209 (9)	0.0240 (9)	0.0212 (9)	0.0018 (7)	0.0021 (6)	0.0005 (7)
C6	0.0210 (9)	0.0206 (9)	0.0187 (8)	0.0009 (7)	0.0016 (6)	−0.0006 (6)
C7	0.0226 (9)	0.0184 (9)	0.0197 (8)	−0.0012 (7)	0.0022 (6)	0.0008 (6)
C8	0.0194 (9)	0.0149 (9)	0.0295 (10)	0.0000 (6)	−0.0012 (7)	0.0010 (6)
C9	0.0220 (9)	0.0222 (9)	0.0265 (10)	0.0007 (7)	0.0001 (7)	−0.0027 (7)
C10	0.0261 (10)	0.0314 (11)	0.0318 (10)	−0.0026 (8)	0.0028 (8)	0.0030 (8)
C11	0.0204 (9)	0.0211 (10)	0.0323 (10)	0.0008 (7)	−0.0066 (7)	0.0002 (7)
C12	0.0252 (9)	0.0268 (11)	0.0367 (11)	−0.0050 (7)	−0.0082 (8)	0.0024 (8)
C13	0.0229 (10)	0.0591 (15)	0.0384 (12)	0.0080 (9)	−0.0058 (8)	0.0033 (10)

### Geometric parameters (Å, °)

**Table:**

S1—C8	1.6663 (18)	C5—C6	1.396 (3)
O1—C7	1.237 (2)	C5—H5A	0.9300
O2—C3	1.367 (2)	C6—C7	1.490 (2)
O2—C13	1.428 (3)	C9—C10	1.514 (3)
N1—C7	1.351 (2)	C9—H9A	0.9700
N1—C8	1.431 (2)	C9—H9B	0.9700
N1—H1A	0.8600	C10—H10A	0.9600
N2—C8	1.327 (2)	C10—H10B	0.9600
N2—C11	1.470 (2)	C10—H10C	0.9600
N2—C9	1.479 (2)	C11—C12	1.517 (3)
C1—C2	1.394 (3)	C11—H11A	0.9700
C1—C6	1.395 (3)	C11—H11B	0.9700
C1—H1B	0.9300	C12—H12A	0.9600
C2—C3	1.387 (3)	C12—H12B	0.9600
C2—H2A	0.9300	C12—H12C	0.9600
C3—C4	1.397 (3)	C13—H13A	0.9600
C4—C5	1.379 (3)	C13—H13B	0.9600
C4—H4A	0.9300	C13—H13C	0.9600
			
C3—O2—C13	117.56 (17)	N2—C9—C10	112.64 (15)
C7—N1—C8	120.83 (15)	N2—C9—H9A	109.1
C7—N1—H1A	119.6	C10—C9—H9A	109.1
C8—N1—H1A	119.6	N2—C9—H9B	109.1
C8—N2—C11	121.01 (15)	C10—C9—H9B	109.1
C8—N2—C9	123.58 (14)	H9A—C9—H9B	107.8
C11—N2—C9	115.40 (14)	C9—C10—H10A	109.5
C2—C1—C6	121.00 (17)	C9—C10—H10B	109.5
C2—C1—H1B	119.5	H10A—C10—H10B	109.5
C6—C1—H1B	119.5	C9—C10—H10C	109.5
C3—C2—C1	119.34 (17)	H10A—C10—H10C	109.5
C3—C2—H2A	120.3	H10B—C10—H10C	109.5
C1—C2—H2A	120.3	N2—C11—C12	111.73 (14)
O2—C3—C2	124.80 (17)	N2—C11—H11A	109.3
O2—C3—C4	115.01 (17)	C12—C11—H11A	109.3
C2—C3—C4	120.19 (17)	N2—C11—H11B	109.3
C5—C4—C3	119.97 (18)	C12—C11—H11B	109.3
C5—C4—H4A	120.0	H11A—C11—H11B	107.9
C3—C4—H4A	120.0	C11—C12—H12A	109.5
C4—C5—C6	120.81 (17)	C11—C12—H12B	109.5
C4—C5—H5A	119.6	H12A—C12—H12B	109.5
C6—C5—H5A	119.6	C11—C12—H12C	109.5
C1—C6—C5	118.69 (17)	H12A—C12—H12C	109.5
C1—C6—C7	117.74 (16)	H12B—C12—H12C	109.5
C5—C6—C7	123.43 (16)	O2—C13—H13A	109.5
O1—C7—N1	120.50 (16)	O2—C13—H13B	109.5
O1—C7—C6	121.81 (16)	H13A—C13—H13B	109.5
N1—C7—C6	117.60 (15)	O2—C13—H13C	109.5
N2—C8—N1	114.73 (15)	H13A—C13—H13C	109.5
N2—C8—S1	126.08 (14)	H13B—C13—H13C	109.5
N1—C8—S1	119.16 (13)		
			
C6—C1—C2—C3	−0.2 (3)	C1—C6—C7—O1	5.0 (3)
C13—O2—C3—C2	−0.6 (3)	C5—C6—C7—O1	−170.57 (16)
C13—O2—C3—C4	179.54 (17)	C1—C6—C7—N1	−178.57 (16)
C1—C2—C3—O2	179.78 (17)	C5—C6—C7—N1	5.9 (2)
C1—C2—C3—C4	−0.3 (3)	C11—N2—C8—N1	176.34 (14)
O2—C3—C4—C5	−179.75 (16)	C9—N2—C8—N1	−2.8 (2)
C2—C3—C4—C5	0.3 (3)	C11—N2—C8—S1	−1.5 (2)
C3—C4—C5—C6	0.2 (3)	C9—N2—C8—S1	179.41 (13)
C2—C1—C6—C5	0.8 (3)	C7—N1—C8—N2	84.6 (2)
C2—C1—C6—C7	−175.01 (16)	C7—N1—C8—S1	−97.46 (17)
C4—C5—C6—C1	−0.8 (3)	C8—N2—C9—C10	84.7 (2)
C4—C5—C6—C7	174.78 (16)	C11—N2—C9—C10	−94.45 (18)
C8—N1—C7—O1	−4.6 (3)	C8—N2—C11—C12	94.1 (2)
C8—N1—C7—C6	178.96 (15)	C9—N2—C11—C12	−86.69 (19)

### Hydrogen-bond geometry (Å, °)

**Table:**

*D*—H···*A*	*D*—H	H···*A*	*D*···*A*	*D*—H···*A*
N1—H1A···O1^i^	0.86	2.05	2.847 (2)	154

Symmetry codes: (i) −*x*+3/2, *y*−1/2, *z*.
